# Results and Future Perspectives of the Sustainable Anesthesia Project: A Large-Scale, Real-World Implementation Study at the Largest Spanish Private Healthcare Provider

**DOI:** 10.3390/healthcare14030300

**Published:** 2026-01-25

**Authors:** Juan Acha-Ganderias, María del Pino Henríquez-de Armas, Luis Enrique Muñoz-Alameda, Ion Cristóbal, Cristina Caramés, Leticia Moral-Iglesias

**Affiliations:** 1 ESG/Sustainability Department, Quirónsalud Healthcare Network, 28040 Madrid, Spain; juan.acha@quironsalud.es (J.A.-G.); mpino@quironsalud.es (M.d.P.H.-d.A.); 2 Anesthesiology Department, Hospital Universitario Fundación Jiménez Díaz, 28040 Madrid, Spain; lemunoz@quironsalud.es; 3 Corporate Medical and Research Department, Quirónsalud Healthcare Network, 28040 Madrid, Spain; ccarames@quironsalud.es; 4 Equipment and ESG/Sustainability Directorate, Quirónsalud Healthcare Network, 28040 Madrid, Spain

**Keywords:** anesthetic gases, nitrous oxide, desflurane, sevoflurane, isoflurane, sustainability

## Abstract

**Background**: Climate change is a serious threat to global health. The healthcare sector contributes substantially to greenhouse gas (GHG) emissions, with anaesthetic gases being a major source of Scope 1 emissions. We aimed to evaluate the 2024 impact of the Sustainable Anesthesia Project, designed to reduce the environmental footprint of anaesthetic gases by eliminating and/or replacing the most polluting agents (nitrous oxide and desflurane) with more sustainable alternatives (sevoflurane, total intravenous anaesthesia, and regional/local anaesthesia). **Methods**: We conducted a descriptive analysis of anaesthetic gas consumption in 2023 and 2024, as well as a comparison of emissions in tons of CO_2_, the impact on the carbon footprint, and the potential future emissions savings that full implementation of the project would entail. **Results**: In the first year, nitrous oxide consumption decreased by 64% and desflurane by 63%. Overall anaesthetic-gas emissions fell by 8386 tCO_2_e versus 2023, a 54% relative reduction. Furthermore, the contribution of these gases to the total Scope 1 emissions markedly declined from 35.18% in 2023 to 21.22% in 2024. An additional reduction potential of around 4800 tCO_2_e was identified for consolidation by 2025 with full implementation. **Conclusions**: The results observed in this study demonstrate the success of the Sustainable Anesthesia Project, whose strategy represents an extensible and applicable option to other centers and companies in the health sector to reduce their environmental impact.

## 1. Introduction

Climate change is now the leading environmental concern, followed by pollution and, in many parts of the world, drought. Greenhouse gas emissions have been recognized as the main cause of climate change and global warming. Global warming potential (GWP) is a measure of how much a greenhouse gas contributes to global warming over a specific period, the international standard being 100 years. The GWP compares a gas’s contribution to an equivalent mass of carbon dioxide, which serves as the unit of comparison. Of note, these factors are updated periodically as atmospheric chemistry is constantly changing [1]. The healthcare sector contributes significantly to greenhouse gas (GHG) emissions, with the WHO estimating that they account for around 4–5% of annual net global emissions. This is equivalent to the emissions of 514 coal-fired power plants, or in other words, if the healthcare sector were a country, it would be the fifth-largest emitter on the planet. In Spain, the Spanish Society of Anesthesia, Resuscitation, and Pain Therapy (SEDAR) indicates that hospitals generate 1% of a country’s solid waste and emit 2.1% of its annual greenhouse gases. The anesthetic process is responsible for 25% of surgical suite waste, with an annual contribution to global warming equivalent to that of one million cars [2]. Therefore, anesthetic gases represent one of the main sources of greenhouse gas emissions in the health sector during its activities (Scope 1), making it necessary to develop initiatives that contribute to minimizing their environmental impact.

Among anesthetic gases, nitrous oxide (N_2_O) and halogenated volatile anesthetics (especially desflurane) have a high climate impact [3]. Nitrous oxide is a widely used anesthetic gas that, in addition to being a GHG, also damages the stratospheric ozone layer [4]. Once in the stratosphere, N_2_O decomposes, releasing nitrogen oxides (NO), which react with ozone (O_3_), catalyzing its destruction and forming nitrogen dioxide (NO_2_). It remains in the atmosphere for over a hundred years, and its GWP is very high. Due to its low anesthetic potency, it requires high concentrations (50–70% mixed with oxygen), which means releasing large quantities per procedure. Furthermore, halogenated volatile anesthetics such as desflurane, sevoflurane and isoflurane contribute to the greenhouse effect by exhibiting high infrared absorption [5]. Moreover, the atmospheric lifetime of these gases varies considerably between desflurane (14 years), isoflurane (3 years) and sevoflurane (2 years). In terms of global warming potential (GWP), desflurane has the highest, followed by isoflurane and sevoflurane. Moreover, desflurane has a low anesthetic potency, which requires its administration at concentrations several times higher than other volatiles, and isoflurane is a chlorofluorocarbon that also damages the ozone layer because it contains chlorine. The above differences imply that for each general anesthesia procedure, the choice of anesthetic gas significantly influences the carbon footprint. In clinical settings, it has been estimated that emissions from desflurane or N_2_O can be up to 40 times higher than those using sevoflurane at similar flow rates [6].

To address this problem and within the framework of its Energy Efficiency, Adaptation, and Climate Change Mitigation Plan, the private healthcare provider Quirónsalud launched its innovative Sustainable Anesthesia Project, an initiative that seeks to minimize the environmental impact of its hospitals by reducing CO_2_ emissions from the use of anesthetic gases. Furthermore, this project is a key component of Quirónsalud environmental strategy, aligned with the goal of reducing Scope 1 and 2 CO_2_ emissions by 50% by 2030 and achieving climate neutrality by 2040. The Sustainable Anesthesia Project is based on two main axes. First, the replacement of anesthetic gases with a high environmental impact (Nitrous Oxide and Desflurane) with less polluting alternatives such as total intravenous therapy, regional and/or local anesthesia and Sevoflurane as the gas of choice in case of inhalation anesthesia. Second, the implementation of low-flow fresh gas anesthesia techniques when inhalation anesthesia is the most appropriate option from the point of view of patient safety and quality of care.

In this work, we show the results of the Sustainable Anesthesia Project, whose results in the first year of its implementation with a reduction of 8386 tCO_2_e in anesthetic gas emissions, reflect the success of this initiative and constitute a reference point for its adoption by other healthcare providers both in Spain and internationally.

## 2. Materials and Methods

### 2.1. Study Design

A retrospective longitudinal observational study examining the evolution of Scope 1 emissions of anesthetic gases during the years 2023 and 2024 was performed. This study included 59 Quironsalud’s hospitals and medical centers from Spain and Colombia to evaluate the effectiveness of the Sustainable Anesthesia Project.

### 2.2. Data Collection and Analysis

This study used indirect data on anesthetic gas consumption obtained from the Purchasing Department and the Corporate Pharmacy through purchases of the various gases at each center. Those Quirónsalud centers with available data during the study period were included. All centers with available data were analyzed, so a sample size calculation did not apply in this study. The implementation of the Sustainable Anesthesia Project was staggered across participating centers, with each progressing through the phases based on local readiness and infrastructure. In the case of the four public hospitals in Madrid, nitrous oxide consumption was already null in three centers by Q1 2024 and in all four by Q2 2024, reflecting the impact of prior institutional efforts. As a result, the most substantial reductions in anesthetic gas use attributable to the project were observed in the private hospital network. The adoption of low-flow anesthesia techniques was promoted through training and internal audits, and safety monitoring—including adverse events and clinical incidents—was conducted under the supervision of the project’s clinical governance committee.

### 2.3. Parameters and Measurements

This study included consumption data for the following anesthetic gases: Nitrous oxide, Desflurane, Sevoflurane and Isoflurane. To calculate the emissions in tCO_2_e for each anesthetic gas, we followed a multi-step process. First, we obtained the annual consumption of each gas in volume units (liters or milliliters) from supplier data. These volumes were converted to mass (kilograms) using the density values provided in the official product data sheets. Next, we adjusted the mass of each gas to account for its estimated metabolism in the human body, based on published pharmacokinetic data. Finally, we applied the 100-year Global Warming Potential (GWP100) coefficients from the IPCC Fifth Assessment Report (AR5) [7] to convert the mass of each gas (after metabolism adjustment) into CO_2_ equivalents. The resulting tCO_2_e values were then aggregated to estimate total emissions per gas and per year. This methodology ensures consistency with international climate reporting standards and enhances the comparability of our results. We performed a descriptive analysis of the different variables under study. Furthermore, we also analyzed the impact of the project on the group’s carbon footprint.

### 2.4. Description of the Project Phases

The design of the Sustainable Anesthesia Project included a series of phases in its implementation, which are detailed in Table 1.

## 3. Results

### 3.1. Characteristics and Environmental Impact of the Anesthetic Gases

We first explored the polluting effect of each of the four anesthetic gases included in the study, as well as their degree of metabolism, which ultimately determines how much of the administered gas is released into the atmosphere. Both parameters are key to determining the emissions derived from the use of each of them.

Regarding its degree of metabolism, the data available in its data sheet indicate that nitrous oxide does not undergo any metabolism [8]. Sevoflurane is poorly soluble in blood and tissues, causing a rapid alveolar concentration sufficient to produce anesthesia and rapid subsequent elimination. In humans, less than 5% of absorbed sevoflurane undergoes hepatic metabolism to hexafluoroisopropane (HFIP), which is conjugated with glucuronic acid and eliminated in the urine [9]. Desflurane produces a rapid induction of anesthesia without metabolism in the liver or other organs and with minimal accumulation in adipose tissue, with an estimated minimum metabolism of 0.02% [10]. Finally, only 0.17% of absorbed isoflurane can be recovered as urinary metabolites [11]. The poor metabolism of these gases indicates that virtually all of the anesthetic gas administered to the patient returns to the atmosphere in its initial composition, thus maintaining its high heating power.

The polluting effect of gases is measured using the GWP 100 factor, which measures how many times more the gas pollutes than CO_2_ with the same amount of mass, expressing the result in Kg of CO_2_ emitted (KgCO_2_e) per Kg of gas (KgCO_2_e/Kg). Of note, Table 2 shows that Desflurane is 8 times more polluting than Sevoflurane, and almost 4 times more than Isoflurane, although the latter contains chlorine and therefore also damages the ozone layer.

Therefore, the two aspects that fundamentally cause the high tCO_2_e emission of anesthetic gases are their high heating power and the poor metabolism of the volume of gas administered to the patient.

### 3.2. Analysis of Suppliers and Product Delivery Formats

An analysis was conducted to understand the diversity of suppliers the company works with, as well as the formats in which the anesthetic gases included in the study are dispensed. Thus, the supply of anesthetic gases to hospitals is carried out by five suppliers, and the service provision is supported by corporate contracts. Table S1 shows the type of gas and its presentation supplied by each supplier.

It should be noted that in the case of using nitrous oxide together with oxygen, the products provided by the suppliers contained a prepared mixture of nitrous oxide (N_2_O) and oxygen (O_2_), in a proportion of 50% of each.

### 3.3. Comparative Analysis of Anesthetic Gas Emissions

Next, we calculated the tons of CO_2_ emitted in 2023 and 2024 for each of the gases. These calculations used consumption data and the respective GWP100 conversion factors, and the results were then adjusted according to their metabolism data (Table 2). In the specific case of sevoflurane, the calculations were made considering a 3% metabolism based on official data published in the report on inhalational anesthetic gases published in the Public Health Commission held by the Interterritorial Council of the Spanish National Health System [12].

The CO_2_ equivalent emissions were calculated by converting the volume of anesthetic agents used into mass (kg) using their respective densities, and then applying the GWP100 values as published by the IPCC. The following formula was used: CO_2_e (kg) = Volume (L) × Density (g/mL) × (1 − Metabolism Rate) × GWP100/1000. Firstly, since the consumption data for sevoflurane, desflurane and isoflurane were obtained in volume units, the density data included in their respective data sheets were used to calculate their weight in kilograms and thus be able to perform the subsequent conversion (Table 3).

The equivalent amounts of CO_2_ emitted for each of the gases were then obtained by adjusting the consumption with their respective metabolism rates and subsequently applying the GWP100 conversion factors (Table 4).

Finally, with the emission data obtained for each gas, a comparison was made between 2023 and 2024, calculating the percentage of global interannual variation and for each of the anesthetic gases (Table 5).

Thus, the 2024 results reflect significant progress in reducing the use of the most polluting anesthetic gases. A 64% reduction in nitrous oxide consumption was observed, from 9110 tCO_2_e in 2023 to 3279 tCO_2_e in 2024, and a 63% decrease in more than 2500 tCO_2_e in the case of desflurane. Furthermore, a similar sevoflurane consumption and a 2% increase in isoflurane consumption were found, resulting in a combined increase in only 9 tCO_2_e. Globally, in 2024, emissions from anesthetic gases were reported to have decreased by 54% compared to 2023, with a total reduction of 8386 tCO_2_e.

To ensure that the observed reduction in anesthetic gas emissions was not influenced by changes in surgical activity, we analyzed the total number of surgical procedures performed in 2023 and 2024 using APR-DRG version 38. The number of ambulatory major surgeries increased from 188,230 in 2023 to 211,159 in 2024, while surgical hospitalizations remained stable (204,654 in 2023 vs. 200,945 in 2024). These data confirm that the emissions reduction is not attributable to a decrease in surgical volume.

Additionally, the distribution of pediatric versus adult patients remained consistent across both years. In 2023, 6.6% of ambulatory surgeries and 4.9% of surgical hospitalizations involved patients under 18 years old, compared to 6.0% and 4.5%, respectively, in 2024. These small differences do not indicate a significant change in case mix.

To further normalize emissions data, we calculated the emissions per surgical procedure. The total anesthetic gas emissions per 100 surgeries decreased from 3.95 tCO_2_e in 2023 to 1.73 tCO_2_e in 2024, representing a 56.2% reduction. This reinforces the conclusion that the observed environmental improvements are attributable to the implementation of the Sustainable Anesthesia Project.

### 3.4. Time Evolution of Emissions During the Year of Project Implementation

**Figure 1:**
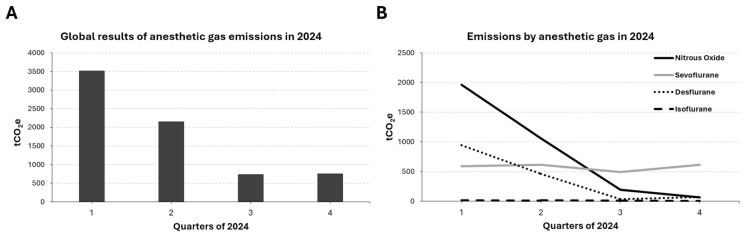
Global (**A**) and gas type-specific (**B**) evolution of emissions in 2024.

In order to further study the effects of the implementation of the Sustainable Anesthesia Project, we analyzed the temporal evolution by quarter of emissions derived from anesthetic gases in the year 2024. As expected, a marked reduction in emissions is observed from the second quarter both in the global analysis and by type of gas (Figure 1 and Table 6).

Finally, the results obtained for 2024 highlight that there is still an additional reduction capacity of almost 4800 tCO_2_e in 2025 if the Sustainable Anesthesia Project is fully implemented, which will lead to the complete elimination of the most polluting anesthetic gases (nitrous oxide and desflurane).

### 3.5. Impact of the Project Implementation on the Group’s Carbon Footprint

**Figure 2:**
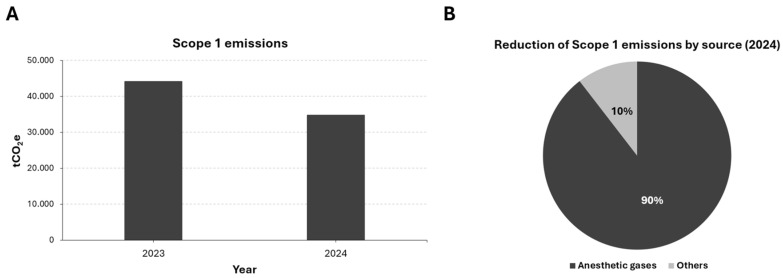
Scope 1 emissions in 2023 and 2024. Absolute emission values (**A**); Proportion of the 2024 emission reduction due to anesthetic gases (**B**).

Regarding the group’s carbon footprint for Scope 1 emissions, a 21.27% decrease was observed, equivalent to a reduction in emissions of 9365 tCO_2_e. Next, we analyzed the impact of the Sustainable Anesthesia Project on this reduction and found that 89.55% of this decrease in emissions was due to the effects derived from the project’s implementation (Figure 2).

To further analyze these data, we separately evaluated the impact of anesthetic gas emissions on Scope 1 emissions in 2023 and 2024. As expected, the contribution of these gases to the total Scope 1 emissions decreased dramatically in 2024 compared to 2023, from 35.18% to only 21.22% of the total (Figure S1).

## 4. Discussion

Here we show the results derived from the implementation of the Sustainable Anesthesia Project, which demonstrate that in less than a year since its launch, the project was able to generate a significant reduction in the Group’s carbon footprint. In 2023, anesthetic gases represented 35% of Quirónsalud’s direct activity emissions (Scope 1) and in 2024 a reduction of up to 21% could be observed (Figure S1), due to a 54% reduction in emissions due to anesthetic gases (equivalent to more than 8300 tCO_2_e) (Table 5). To accelerate its implementation in March 2024, the initiative was presented to all the group’s Medical Directorates a month earlier, and a Technical Committee was created to promote and monitor it (Table 1). Through initiatives like this, Quirónsalud reaffirms its leadership in the Spanish healthcare sector, focusing on healthcare excellence, technological innovation, and sustainability as fundamental pillars of its business.

Furthermore, the study results have shown an additional reduction capacity of approximately 4800 tCO_2_e that could be achieved with the full implementation of the project, which would lead to a total reduction in nitrous oxide and desflurane consumption. In this regard, the time evolution of consumption during 2024 shows a drastic reduction in reported nitrous oxide consumption, as well as a total elimination of desflurane use starting in the third quarter (Table 3). In addition, to strengthen the application of the low-flow fresh gas technique, the company has progressively upgraded its anesthesia tower fleet, adding more than 90 new units by 2024. This upgrade will improve digitalization, safety, and efficiency in clinical practice. The transition toward alternative techniques such as TIVA and regional anesthesia has been shown to be safe and well-tolerated in clinical practice [1,2,3,4]. Numerous studies have demonstrated that these methods offer comparable or even superior outcomes in terms of intraoperative hemodynamic stability, reduced postoperative nausea and vomiting, faster recovery times, and improved pain control [13,14,15,16,17]. Importantly, the Technical Committee specifically created for the monitoring and follow-up of the project did not report any adverse care events related to patient safety within the scope of the Sustainable Anesthesia Project. Furthermore, the use of regional anesthesia and multimodal analgesia strategies contributed to high levels of patient satisfaction, as they allowed for effective pain management while minimizing the use of opioids and avoiding the environmental impact of volatile agents [15,18]. These findings support the clinical viability of sustainable anesthesia practices without compromising patient safety or comfort.

Data from the literature show that in recent years, various hospitals and hospital networks have been working to reduce emissions derived from their activities, and more specifically, those produced by the use of anesthetic gases. Similarly to the Sustainable Anesthesia Project analyzed in this study, various initiatives have also been reported aimed at eliminating and/or replacing the most polluting ones (N_2_O, desflurane) with more sustainable alternatives (sevoflurane, total and regional intravenous anesthesia). Thus, the University of Michigan Health System launched the Green Anesthesia Initiative (GAIA) in 2022 with the goal of reducing the use of N_2_O and desflurane, promoting sevoflurane and TIVA. A retrospective study of more than 90,000 surgeries [19] compared the year before and the year after GAIA and found a halving of CO_2_e emissions. Notably, these results would be comparable to the 56% emissions reduction reported by our study (Table 5). Moreover, the English National Health Service has developed an aggressive plan in recent years to eliminate desflurane from its hospitals, given its high environmental impact. Between 2018 and 2023, the NHS reduced desflurane use from >20% to ~3% of all inhalational anesthesia, with many hospitals phasing it out entirely [20]. In Germany, a before-and-after trial evaluated the effect of removing desflurane vaporizers and informing staff about their environmental impact, without completely banning their use, in a tertiary hospital (32 operating rooms). Desflurane consumption fell dramatically, with no significant increase in sevoflurane or propofol use, indicating that many cases were converted to regional anesthesia or TIVA. No changes in clinical outcomes were observed, and costs also decreased [6]. The authors note that desflurane lacks sufficient clinical benefits to justify its enormous environmental and economic cost, given that sevoflurane offers similar efficacy in most patients. In Spain, a hospital reported implementing a program in 2020 and 2021 to achieve net-zero anesthetic gas emissions in operating rooms by eliminating the use of N_2_O and installing a gas capture and recycling system in anesthesia circuits to capture exhaled desflurane and sevoflurane and optimizing low fresh flows [21]. This program successfully reduced anesthetic gas emissions to zero measurable levels in the surgical environment. Altogether, these studies show that simple measures such as removing vaporizers (requiring them to be requested if you want to use them) and educating about carbon footprint can achieve very rapid and substantial reductions. Within the Quironsalud group, the Fundación Jiménez Diaz University Hospital eliminated the use of nitrous oxide in 2023, prioritizing total intravenous anesthesia (TIVA) and regional/local anesthesia. Furthermore, the use of sevoflurane as the anesthetic gas of choice for inhalational anesthesia and the use of low-flow techniques have been encouraged. This same initiative has been implemented at the beginning of 2024 by the other three public Quironsalud hospitals in Madrid. In addition, ten other hospitals in the group’s private network have implemented various initiatives aimed at limiting the use of nitrous oxide and/or desflurane. Thus, the Sustainable Anesthesia Project has led a corporate initiative to centralize sustainability actions for anesthetic gases at the company level, ensuring that all hospitals are aligned according to this strategic line.

Our results are consistent with published meta-analyses showing that multifaceted ‘green anesthesia’ initiatives can significantly reduce anesthetic gas emissions. Hammer et al. reported a 68% per-anesthetic reduction after interventions like eliminating desflurane, low-flow techniques, and staff education [22]. It is also important to highlight that the transition to low-PCA anesthetics can be economically beneficial. For example, Lehmann et al. [6] observed that eliminating desflurane resulted in substantial annual cost savings without compromising clinical care. Similarly, other institutions report savings of tens of thousands of dollars annually after discontinuing desflurane in favor of more economical alternatives, in addition to a reduction in greenhouse gas emissions [23]. These economic findings support the long-term (clinical and financial) sustainability of “green anesthesia” initiatives.

Furthermore, eliminating nitrous oxide and desflurane has not been shown to harm patient outcomes when replaced by regional anesthesia or other techniques. In fact, large studies and reviews indicate no increase in mortality or major complications after removing these gases [6,19,24,25]. Together, these data provide strong long-term justification—both clinical and economic—for an institutional decision to phase out N_2_O and desflurane in pursuit of sustainable anesthesia. Regarding pain management, the work by Breivik et al. [26] emphasized multidimensional pain assessment for a more complete picture of analgesic efficacy. The authors claimed that valid pain evaluation should include functional outcomes and qualitative descriptors, since true “objective” measurement of pain is impossible. In line with current guidelines, postoperative pain was managed with a multimodal analgesia approach (including regional anesthesia and non-opioid analgesics) to ensure adequate pain relief despite avoiding N_2_O and desflurane. Pain assessment did not rely solely on the visual analogue scale. Postoperative pain should be assessed not just by intensity (e.g., VAS/NRS scores), but also by its impact on recovery (e.g., ability to mobilize, need for rescue analgesics) and patient satisfaction [26]. Moreover, clinical guidelines (e.g., the APS/ASA postoperative pain guideline) recommend multimodal analgesia—combining regional anesthesia and non-opioid analgesics—to maintain high-quality pain relief while minimizing opioids, highlighting that sustainability initiatives do not sacrifice patient comfort or pain management quality [27,28,29].

A major limitation of this study is that it does not consider the potential CO_2_ emissions associated with the production, packaging, and disposal of the drugs and materials used in TIVA. While the reduction in volatile anesthetic gases represents a clear environmental benefit, a comprehensive life cycle assessment, including the carbon footprint of TIVA components, would be necessary to accurately quantify the net environmental impact of this transition. Future studies should incorporate these factors to provide a more complete evaluation of sustainable anesthesia strategies. Furthermore, this study used a non-probability convenience sampling approach, including only centers with available and complete data during the study period. While this method allowed for the practical implementation of a large-scale, real-world intervention, it may introduce selection bias and limit the generalizability of the findings. We acknowledge this as a limitation and recommend that future studies aim for more representative sampling strategies to enhance external validity. Another limitation of this study is its external validity. The intervention was implemented in a specific group of hospitals belonging to the same healthcare network, which could limit the generalizability of the findings to other institutional or national contexts. Differences in infrastructure, clinical protocols, and resource availability between healthcare systems could influence the feasibility and impact of similar interventions elsewhere. In addition, the absence of detailed clinical indicators in our study highlights the need for future prospective studies including structured clinical outcome data, including anesthesia duration, recovery times, and adverse event tracking as well as patient-level data to enable more rigorous statistical modeling. Notably, it should be noted that this study analyzes indirect consumption data, and the information comes from delivery notes and invoices from gas suppliers for supplies made to the hospitals, rather than from actual consumption, the measurement of which would require the introduction of complex monitoring systems. This is a significant limitation, as estimating anesthetic gas consumption using purchasing data may not accurately reflect actual clinical use. While this approach allowed for consistent data collection across multiple centers, it could be influenced by factors such as stockpiling, waste, or variations in ordering practices. Future studies should incorporate direct consumption data from anesthesia machine logs or flowmeters to validate and refine these estimates. Another limitation is that each supplier provides information at different intervals and with their own units of measurement. Finally, it should also be noted that data capture is highly manual, which could affect the accuracy of the information.

## 5. Conclusions

In conclusion, anesthetic gases are one of the main sources of greenhouse gas emissions in the healthcare sector, and there is an urgent need within the sector to implement initiatives that contribute to reducing these emissions. Therefore, the results obtained highlight Quirónsalud’s Sustainable Anesthesia project as a successful strategy and an extensible and applicable option that could help other centers in the sector achieve similar results that contribute to decreasing their environmental impact.

## Figures and Tables

**Table 1:** Description of each of the different development phases of the Sustainable Anesthesia Project.

Phase	Description
I	Presentation of the sustainable anesthesia project to medical directors.
II	Presentation of the project by the medical directors to the anesthesia services of the hospitals.
III	Feedback to the ESG department from medical directorates regarding potential problems in the implementation of project measures and achievement milestones specific to each center.
IV	Creation of a Technical Committee to support project implementation. This committee will address controversial issues related to anesthetic gases and clinical practice and will monitor consumption trends on a quarterly basis for the first year of implementation.
V	Eliminate the use of Nitrous Oxide in all the hospitals.
VI	Prioritize the use of Sevoflurane in case of inhalational anesthesia, progressively eliminating the use of Desflurane.
VII	Apply the low fresh gas flow technique in all inhalational anesthesia.
VIII	Promote the use of non-inhalational anesthesia, such as Total Intravenous Anesthesia (TIVA), regional and/or local anesthesia, whenever clinically feasible and safe for the patient.

**Table 2:** Comparative analysis of the characteristic and environmental impact of the anesthetic gases included in the study.

Gas	Chemical Formula	GWP 100 *	Metabolization
Nitrous Oxide	N_2_O	265	0%
Sevoflurane	C_4_H_3_F_7_O	216	<5%
Desflurane	C_3_H_2_F_6_O	1790	0.02%
Isoflurane	C_3_H_2_ClF_5_O	491	0.17%

* IPCC 5-GWP 100: 100-year Global Warming Potential, which measures the kilograms of CO_2_ emitted per kilogram of anesthetic gas (KgCO_2_e/kg).

**Table 3:** Calculation of anesthetic gas consumption in the years 2023 and 2024.

Gas	Cons. in 2023 (mL)	Cons. in 2024 (mL)	Density (g/mL)	Cons. in 2023 (kg)	Cons. in 2024 (kg)
Sevoflurane	7,208,107	7,233,532	1.52	10,956	10,995
Desflurane	1,548,208	573,433	1.47	2276	843
Isoflurane	60,269	61,870	1.50	90	93

Cons.: consumption.

**Table 4:** Calculation of CO_2_ emission levels by each gas in the years 2023 and 2024.

Gas	Cons. in 2023 (kg)	Cons. in 2024 (kg)	Not Metab.	GWP100 (kgCO_2_e/kg)	tCO_2_e. 2023	tCO_2_e. 2024
Nitrous Oxide	34,377	12,373	100%	265	9110	3279
Sevoflurane	10,956	10,995	97%	216	2296	2304
Desflurane	2276	843	99.98%	1790	4073	1509
Isoflurane	90	93	99.83%	491	44	45

Cons.: consumption; Not Metab.: not metabolized; tCO_2_e: tons of CO_2_ emissions.

**Table 5:** Comparison of anesthetic gas emission levels between the years 2023 and 2024.

CO_2_ Emission Levels (tCO_2_e) *			
Gas	Year 2023	Year 2024	Variation
Nitrous Oxide	9110	3279	−64.01%
Sevoflurane	2296	2304	+0.34%
Desflurane	4073	1509	−62.96%
Isoflurane	44	45	+2.27%
Total	15,523	7137	−54.02%

* tCO_2_e: tons of CO_2_ emitted.

**Table 6:** Quarterly evolution of emissions by anesthetic gases in 2024.

Gas	Q12024 (tCO_2_e)	Q2 2024 (tCO_2_e)	Q3 2024 (tCO_2_e)	Q4 2024 (tCO_2_e)	Total 2024 (tCO_2_e)
Nitrous Oxide	1963	1060	192	65	3279
Sevoflurane	587	613	489	614	2304
Desflurane	944	461	36	68	1509
Isoflurane	15	14	10	6	45
Total	3,509	2,148	727	753	7137

Q: quarter; tCO_2_e: tons of CO_2_ emissions.

## Data Availability

The data underlying this article are available in the article and in its online Supplementary Material.

## Supplementary Materials

The following supporting information can be downloaded at: https://www.mdpi.com/article/10.3390/healthcare14030300/s1. Figure S1: Total Scope 1 emissions in the years 2023 (A) and 2024 (B); Table S1: Presentation provided for each type of gas according to the supplier.
