# Supplementary material for: Results and Future Perspectives of the Sustainable Anesthesia Project: A Large-Scale, Real-World Implementation Study at the Largest Spanish Private Healthcare Provider
Source: Healthcare (Basel). 2026 Jan 25;14(3):300. doi: 10.3390/healthcare14030300 (PMC12896554; doi:10.3390/healthcare14030300)
Supplement: Supplementary file 1 [file healthcare-14-00300-s001.zip › Table S1def.pdf]

Table S1. Presentation provided for each type of gas according to the supplier.

Gas	Supplier	Presentation
Nitrous Oxide	A	Bottle (35 kg)
	B	Bottle (37.5 kg)
	C	Bottle (30 kg or 37.5 kg)
Nitrous Oxide + O2	A	Bottle (5 l or 11 l)
	B	Bottle (5 l)
Sevoflurane	D	Bottle (250 ml)
	E	Bottle (250 ml)
Desflurane	D	Bottle (240 ml)
Isoflurane	D	Bottle (240 ml)
