# Supplementary figures and images for: Results and Future Perspectives of the Sustainable Anesthesia Project: A Large-Scale, Real-World Implementation Study at the Largest Spanish Private Healthcare Provider
Source: Healthcare (Basel). 2026 Jan 25;14(3):300. doi: 10.3390/healthcare14030300 (PMC12896554; doi:10.3390/healthcare14030300)

## Slide 1
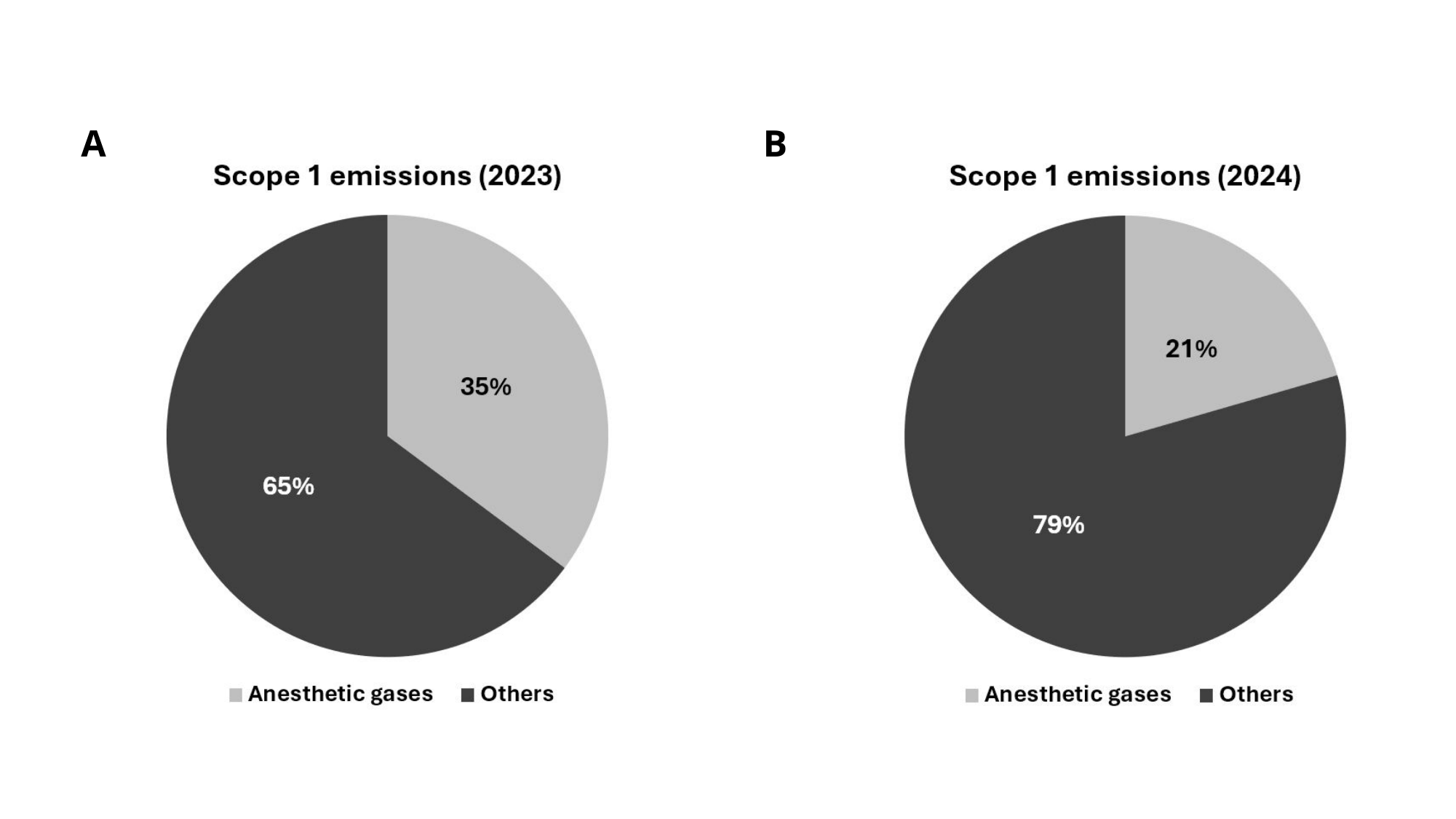

A
B

Supplement: Supplementary file 1 [file healthcare-14-00300-s001.zip › Figure S1.pptx]
